# The Canadian Hospital Association

**Published:** 1911-07-15

**Authors:** 


### THE CANADIAN HOSPITAL ASSOCIATION.

### HOSPITAL PROBLEMS IN THE DOMINION.

The following is a summary of the proceedings at the fifth annual meeting of the Canadian Hospital Association which was held at Niagara Falls, Ontario, on May 23 and 24 last. There was a representative attendance from all parts of the Dominion. The proceedings were opened by the address of the president, Miss C. F. Green, Belleville, Ontario, who dealt in a very able manner with the numerous problems presenting themselves for discussion among hospital workers. Chief among these was the matter of amalgamation with the Superintendent of Training Schools Association and the Graduate Nurses' Association, the main argument for amalgamation being the desire that there was for a Dominion Registration Law in connection with the nursing profession. This matter was subsequently discussed at considerable length and a committee appointed to work out in detail a comprehensive scheme in connection with which they were to report at the next meeting.

Miss Bertha Miller, of St. Thomas, dealt with the management of a small hospital, in which she referred to the relations existing between the Board of Trustees and the management of the hospital, the visiting staff, the training school, the question of securing competent employees, and the best methods of keeping track of supplies and rendering accounts.

Dr. W. J. Dobbie had assigned to him the subject of "Fire Protection in Hospitals," this subject having been suggested as being of timeous interest on account of the fact that during the past few months several hospitals in Canada had had experiences with fire, the most notable being the Hospital for Insane, Manitoba, the Toronto Free Hospital at Weston, and the Hospital for Sick Children, Toronto. After relating briefly the events connected with the destruction of the Toronto Free Hospital in which forty patients were removed in safety, various points in connection with fire protection were discussed. These included the construction of fireproof buildings, fire appliances such as standpipes, hose, fire pails, mechanical fire-extinguishers, fire escapes, etc. The question of fire drill in hospitals was given considerable attention and a comprehensive plan for use in institutions of different sizes outlined.

Dr. W. B. Kendall, Gravenhurst, dealt with the subject of "Sanatoria in Europe," in which he described the methods adopted in various institutions in that country, chief among which were the King Edward Sanatorium, the Sanatorium at Frimley, and also that on the Isle of Wight.

A most instructive paper was that by Dr. Wayne Smith, St. Louis, who described in detail the construction and equipment of a model contagious disease hospital, illustrating by lantern views not only the plans and divisions of the wards, but also many points in connection with the equipment of the same.

The "Heating and Ventilation of Small Hospitals" was very ably dealt with in a paper by Mr. Clarence Williams, of the firm of Williams and Cole, hospital experts, Boston. This paper was read by Mr. Cole and in it were described the various mechanical devices by which the air contained in hospitals could be kept in a state of maximum purity and at a fixed temperature.

An interesting paper was that on the "Relation of Different Hospital Charities" by Mr. B. Burritt. This paper proved to be most interesting in that it showed the close relation that should exist between all charities in any community so that there may be no waste effort or duplication of work in connection with the care of the sick and the destitute. Dr. J. S. Hart, Toronto, dealt with the subject of "What the Average Medical Man Expects from the Hospital," in which he established high ideals which should be performed by the hospital, and of the ethical relations that should exist between the family physician on the one hand and the visiting staff on the other, as well as the relations existing between the hospital and the patient in their relation to the average medical pracitioner.

The officers elected for the ensuing year were: President, Dr. H. A. Boyce, Kingston, Ont.; Vice-Presidents, Miss M. J. E. Morton, Collingwood, Ont.; Dr. Lincoln, Calgary, Alberta; Miss C. F. Green, Belleville, Ont.; Miss Rodgers, Niagara Falls, Ont.; Mrs. Fournier, Gravenhurst, Ont.; Secretary, Dr. J. N. E. Brown, Toronto, Ont.; Treasurer, Miss Mathieson, Toronto, Ont.

